# A Ratiometric Fluorescence Nano pH Biosensor for Live-Cell Imaging Using Cerasome

**DOI:** 10.3390/bios15020114

**Published:** 2025-02-16

**Authors:** Zhongqiao Zhang, Xiaoshan Luo, Xuanbo Wang, Meng Liu, Xiuli Yue, Zhaozhu Zheng

**Affiliations:** 1 School of Food Science and Technology, Jiangnan University, Wuxi 214122, China; 2 National Engineering Laboratory for Modern Silk, Soochow University, Suzhou 215123, China2215408087@stu.suda.edu.cn (X.W.); 3 Cyrus Tang Hematology Center, Collaborative Innovation Center of Hematology, Suzhou Medical College, Soochow University, Suzhou 215123, China; liumeng@suda.edu.cn; 4 School of Municipal and Environmental Engineering, Harbin Institute of Technology, Harbin 150001, China

**Keywords:** ratiometric fluorescence, pH sensor, nanoparticles, cerasome, live cells

## Abstract

The development of a robust and biocompatible pH-sensing platform is critical for monitoring intracellular processes and diagnosing diseases. Here, we present a smart ultrastable ratiometric fluorescence nano pH sensor based on silica-coated liposome nanoparticles (cerasome, 138.4 nm). The sensor integrates pH-sensitive dye, pyranine, within cerasome, achieving enhanced photostability, sensitivity, and biocompatibility. Its unique ratiometric design enables precise pH monitoring with minimal photobleaching and quenching, covering a linear detection range of pH 6.25–8.5. The hybrid nanoparticles exhibit high morphological stability, making them suitable for real-time intracellular pH measurement. This novel platform shows great promise for applications in cellular biology, disease diagnosis, and therapeutic monitoring, offering a versatile tool for biomedical research.

## 1. Introduction

The hydrogen ion concentration (pH) is a fundamental parameter in cellular biology, reflecting metabolic activities, ion transport, and pathological states. Aberrations in intracellular pH are closely linked to various diseases, including cancer, neurodegenerative disorders, and metabolic dysfunctions [1,2]. Articles have reported a connection between diseases and abnormal pH values [3]. Therefore, precise and reliable methods for real-time pH monitoring in live cells are essential for advancing both diagnostics and therapeutics. Nowadays, there is growing interest in the exploration and innovation of (bio)sensors for acidity measurement, particularly in biological fluids and cellular systems [4], pharmaceutical analyses [5], environmental assessment [6], and other fields [7,8]. In this domain, fluorescence-based pH sensors are particularly advantageous due to their high sensitivity, real-time monitoring capabilities, and adaptability to complex biological environments [9,10]. Until now, diverse strategies have been devised for the development of pH sensors. These sensors function by assessing fluorescence intensity [9], the ratio of fluorescence intensities between two emission wavelengths [6,11,12,13], or fluorescence lifetime [1]. The measurement of fluorescence intensity is among the most commonly employed techniques [14,15]. However, many fluorescent dyes, when freely diffused in biological environments, are prone to interactions with other fluorescent entities or biomolecules, such as proteins or cellular metabolites. These interactions can cause photobleaching, instability in biological media, and limited linear detection ranges [16,17,18,19]. Immobilization methods can be used to prevent the free movement of fluorescent dyes in biological environments, thereby enhancing their photostability and functional specificity.

Current dye encapsulation systems can be classified into two categories, (a) silica-based and (b) lipid-based, where dyes are either embedded in or bonded to silica or lipid components. Silica molecules, due to their ability to undergo hydrolysis and self-assembly, are commonly utilized to create solid structures such as silica microspheres, nanoparticles [20,21,22], and films [8,23,24,25]. In contrast, lipids are primarily employed to form liquid or liquid crystalline phases, including phospholipid vesicles (liposomes) [26,27,28,29], micelles, and various emulsions [30]. The silica- and lipid-based systems also have many commonalities in fabrication, analysis, and implementation. Conversely, lipid-based systems frequently encounter stability issues, whereas silica-based systems remain stable; however, lipid-based systems generally exhibit greater biocompatibility than silica-based systems, given that lipids are a fundamental part of the human body. Through the integration of silica-based and lipid-based systems, we recognized the potential to harness their benefits while reducing their limitations, ultimately developing novel smart sensors with improved features for diverse applications.

To address these limitations, hybrid systems that combine the biocompatibility of lipid-based materials with the stability of silica matrices have gained attention. For example, in liposome-in-microsphere (LIM) systems [30], silica coating has been effectively applied to liposomes to enhance stability. Considering that combining silica matrix and liposomes to form silica- and lipid-based systems will be a big challenge, in the present research, we intended to develop a novel dye immobility system. In this novel system, an organic–inorganic hybrid composite was used as nanoparticles forming lipids [31].

Fluorescent dyes are often affected by the presence of other fluorescent molecules, which can lead to fluorescence quenching or interference, compromising the stability and accuracy of measurements. A single fluorescent signal is challenging to utilize effectively because of its susceptibility to photobleaching upon prolonged light exposure or interaction with other fluorescent molecules. To address this, many studies have focused on immobilizing two fluorescent dyes within a single system. This dual-dye approach improves stability, accuracy, and reliability by incorporating one pH-sensitive dye and one stable reference dye. In other systems, Förster resonance energy transfer (FRET) has been utilized to modulate the fluorescent signal, enhancing functionality and overcoming challenges associated with using a single dye. So, in most of the reported works, research on the immobilization of two dyes in one system has been executed, where one is dependent and another is independent of pH or FRET.

In this work, we developed a novel ratiometric nano pH sensor capable of real-time optical detection of pH changes in live cells. The sensor was created by embedding pH-sensitive fluorescent dyes into organic–inorganic hybrid nanoparticles using sol–gel and self-assembly techniques. Its innovative design incorporates a bioinspired colloidal structure with an inner aqueous compartment, similar to a liposome, which enhances its ability to integrate seamlessly into cellular environments. The surface of the nanoparticles is coated with a single molecular layer of inorganic silica, which not only protects the fluorophores from photobleaching but also improves the sensor’s mechanical and chemical stability. This organic–inorganic hybrid structure significantly increases the sensor’s durability, enabling it to provide stable and accurate fluorescence signals over extended periods, even under fluctuating conditions. The ratiometric sensing mechanism, which compares fluorescence intensities at different wavelengths, ensures higher accuracy by compensating for common issues like photobleaching and quenching. The sensor demonstrates high sensitivity, linearity, and a broad detection range, making it an ideal tool for real-time monitoring of pH changes in live cells, with potential applications in cellular biology, biomedical research, and disease monitoring.

## 2. Experimental

### 2.1. Reagents

N,N-dihexadecyl-Nα-(6-((3-triethoxysilyl)propyldimethylammonio) hexanoyl) alaninamide bromide was synthesized according to the method reported in [31]. pH-sensitive dye pyranine (1-hydroxypyrene-3,6,8-trisulfonic acid) and sodium polystyrenesulfonate (PSS, MW 56, 000) were obtained from Sigma-Aldrich [Shanghai, China]. A buffer solution spanning a pH range of 5.5 to 8.5 was formulated with 20 mM Na_2_HPO_4_ [Sigma-Aldrich, Shanghai, China] and KH_2_PO_4_ [Sigma-Aldrich, Shanghai, China]. All other chemicals were of analytical grade. 

### 2.2. Apparatus

The fluorescence was measured by a Varian Cary Eclipse spectrometer (Varian, Palo Alto, CA, USA). Absorption spectra were recorded with a Varian Cary 4000 UV–visible spectrophotometer (Varian, Palo Alto, CA, USA). An Olympus IX71 inverted fluorescence microscope was used for fluorescence imaging (Olympus, Tokyo, Japan). A scanning electron microscope (SEM; Hitachi S-4300, Tokyo, Japan) was used to obtain SEM images. Zeta potential and particle size were analyzed by dynamic light scattering (DLS) (BIC, Brookhaven, GA, USA). All the pH measurements were made with a Thermo Electron Corporation instrument (Model 868, Waltham, MA, USA). A vortex mixer (Vortex-5, Qilinbeier, Nantong, China) was used to obtain multilamellar vesicles (MLVs). A probe-type sonicator (Sonicator 3000, New Milford, CT, USA) was used to obtain nanoparticles.

### 2.3. Preparation of Nano pH Sensor

**Scheme 1:**
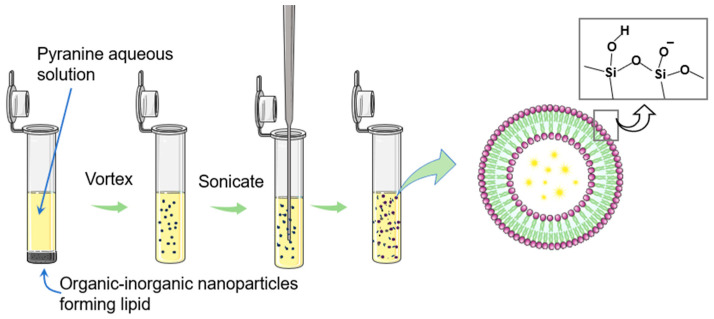
A schematic of the preparation method of the nano pH sensor.

As shown in Scheme 1, 7 mL of 2.28 μM pyranine [31] aqueous solution was added to 3.4 mg of organic–inorganic hybrid lipid (0.4857 mg/mL). The suspension was thoroughly stirred using a vortex mixer until a homogeneous milky consistency was achieved. At this stage, the primary components were multilamellar vesicles (MLVs), averaging a diameter of a few hundred nanometers. Subsequently, the MLV dispersion underwent sonication at 30 W for 3 min in pulse mode (3 s on, 3 s off) using a probe-type sonicator, with temperatures maintained above 23 °C. The sonicated solution was then allowed to rest for a minimum of 12 h at room temperature, enabling hydrolysis to form a silica surface with Si-O-Si networks.

### 2.4. Reversibility and Reproducibility of Nano pH Sensor

According to reports [32,33], these organic–inorganic hybrid nanoparticles can be used for the preparation of three-dimensional (3-D) packed vesicular supramolecular nanodevices by traditional layer-by-layer self-assembly. Quartz substrates were treated with Piranha solution, which is highly corrosive and toxic, for cleaning purposes. The 3-D nanodevices were alternatively deposited from the as-prepared nano pH sensor solution and 1 mg mL-1 PSS aqueous solution (pH 7.5, 0.5 M NaCl), each for 10 min, followed by intermediate rinsing with water and drying under a nitrogen stream. The organic–inorganic hybrid lipid was positively charged. The ξ-potential value of the nano pH sensor was +32.90 ± 1.44 mV at pH 7.0. The electrostatic interaction between negative PSS and positive nanoparticles provided a powerful charged nano-environment in the film, which greatly improved the stability of the 3-D structure.

The nanodevice was put into a modified hermetic quartz cuvette with a home-made solid sample holder and liquid in/out pass. The buffer was changed by injection and fluorescence kinetics measurements were taken at Ex 413 and 460 nm and Em 513 nm.

### 2.5. Nano pH Sensor Uptake by Cells and Intracellular pH Measurement

HeLa cells (Shanghai Academy of Life Sciences and Chinese Academy of Sciences, Shanghai, China) were plated at a density of 3 × 10^4^ per well in 24-well plates and incubated for 24 h in RPMI 1640 medium (Gilbco, MA, USA) containing 10% fetal bovine serum (FBS, Hyclone, UT, USA) and 1% antibiotic (Pen-Strep, Aladdin Reagent Company, Shanghai, China). After replacement with 1 mL RPMI 1640 medium (Hyclone, UT, USA) containing 20 μL of the prepared nano pH sensors, the cells were incubated at 37 °C with 5% CO_2_ for 3 h. Before fluorescence observations, cells were washed with PBS (pH 7.5) three times. For intracellular pH measurement, 10 μL HCl (0.1 M) was added before capturing images.

### 2.6. Sample Analysis

First, adherent cells were detached using 0.25% trypsin, and 50 μL of the prepared nano pH sensors was added and incubated with cells for 3 h at 37 °C and 5% CO_2_, shaking the samples every 30 min. The cells were rinsed using PBS solutions with different pH values (6.7, 7.5, 8.0). Fluorescence measurements were taken at Ex 413 nm and 460 nm, and Em 512 nm. The calibration curve was utilized to assess the pH values of the samples.

## 3. Results and Discussion

### 3.1. Nano pH Sensor Characterization

**Figure 1:**
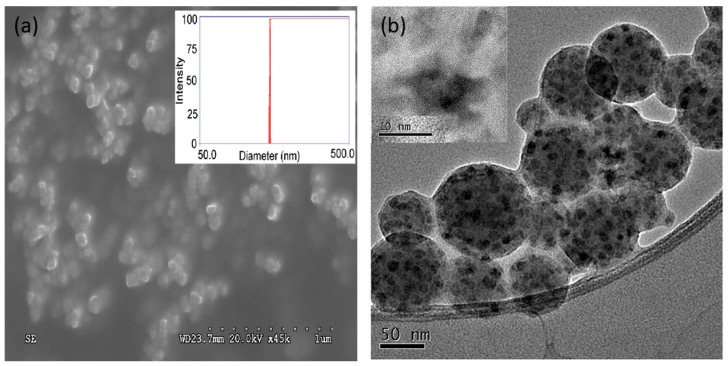
Particle size and morphology characterization of nano pH sensor. (**a**) SEM image of the nano pH sensor, illustrating its structural stability and uniform morphology. The inset in the upper left corner shows the DLS measurement, confirming a consistent average particle size of 138.4 ± 4.5 nm. (**b**) TEM image of the nano pH sensor, revealing the encapsulation of the dye molecules within the liposome core and the silica shell structure surrounding the nanoparticles.

The sol–gel method yielded uniform organic–inorganic hybrid fluorescent nanoparticles. The nanoparticles were characterized with DLS, microscopy, and spectroscopic methods. DLS measurements showed that the nanoparticles had consistent sizes, with an average diameter of 138.4 ± 4.5 nm (Figure 1a insert). According to the SEM observations in Figure 1a, the nano pH sensor maintained its structural integrity and showed no signs of destruction when exposed to the environment. Although vesicular structures are not directly observed in the SEM images, their presence is supported by the nanoparticle preparation method and complementary characterization techniques such as DLS. To visually confirm the encapsulation, we conducted TEM analysis of nanoparticles. As shown in Figure 1b, dye aggregate was dispersed in the middle of the liposome, rather than the aggregate on the surface of the silica shell, to form a core–shell structure. Keeping the pH sensor suspension at room temperature for several months led to no observable changes (image). The morphology of the sensor was observed, and no aggregation was detected (taken by DLS). The morphological stability of the nanoparticles against the micellar-forming surfactant was drastically increased compared with that of the conventional liposomes. The morphological stability was primarily governed by the siloxane networks formed on the vesicular surface, with the hydrogen-belt domain established among the linking units in the nanoparticles contributing supplementary stability.

**Figure 2:**
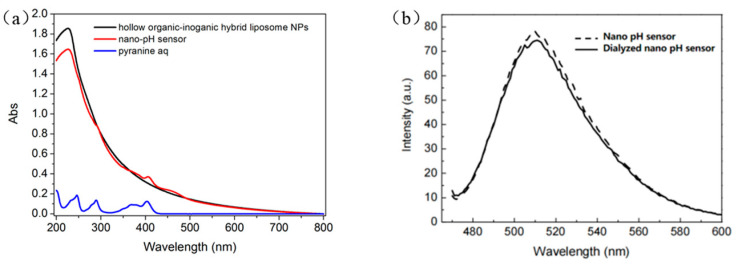
Spectral characterization of nano pH sensor. Ultraviolet–visible absorption spectra of pyranine unloaded nanoparticles (dark), nano pH sensor (red), and free pyranine dye (blue) (**a**). Fluorescence spectra of nano pH sensor before and after dialysis (**b**).

Similar to natural liposome vesicles, these synthetic vesicles are capable of encapsulating guest molecules within their core. We chose the water-soluble pH-sensitive dye as a guest molecule to explore the possible information transfer in loaded vesicles. Spectrofluorometric measurements were performed to characterize the pH sensor. In aqueous solution, the UV-Vis absorption spectra for the pure dye, pH sensor, and unloaded nanoparticles were measured; 2 mL of the prepared nano pH sensor suspension was added to a 3500 D dialysis bag, and 5 L of pure water was used as the dialysate for 48 h. We changed the dialysate every 6 h, removed the nano pH sensor suspension from the dialysis tubing, and measured the UV-Vis absorption spectrum and fluorescence spectrum at 460 nm. Pure pyranine and the pH sensor showed an absorption peak at 405 nm (Figure 2a), which was in line with previously reported values [34,35]. As can be seen from this Figure 2b, the nano pH sensor has a high embedding efficiency of 96.43% for pyranine. The molar ratio of unloaded nanoparticles to pyranine molecules in the nano pH sensor was as high as 442.5. Since nanoparticles form lipids with a positive charge, the pyranine molecule is also positively charged, so the pyranine molecule can be embedded in the aqueous phase surrounded by the lipid bilayer and can also be adsorbed on the inner and outer surfaces of the lipid bilayer through electrostatic action, which makes the nano pH sensor have a high encapsulation efficiency. Thus, pyranine molecules were successfully encapsulated in the aqueous phase of these lipid bilayers.

### 3.2. Application of the Nanoparticles for pH Sensing

**Figure 3:**
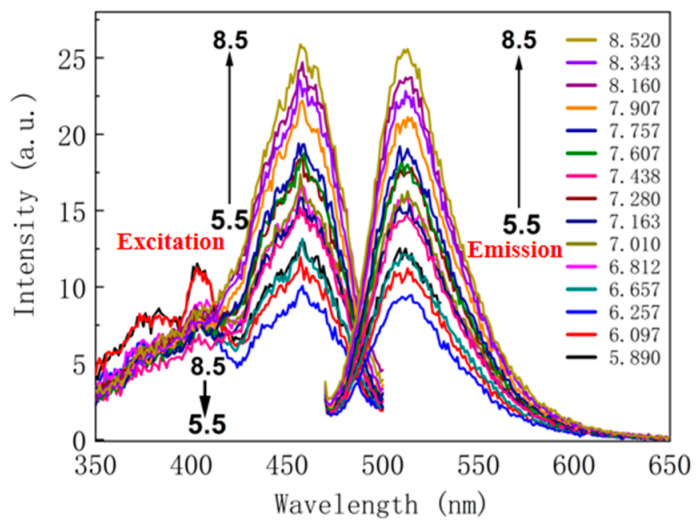
Excitation (**left**, Ex 350–500 nm, Em 513 nm) and emission (**right**, Ex 460 nm, Em 470–650 nm) spectra of nano pH sensor in PBS at different PH values; pH value: 5.5–8.5.

**Figure 4:**
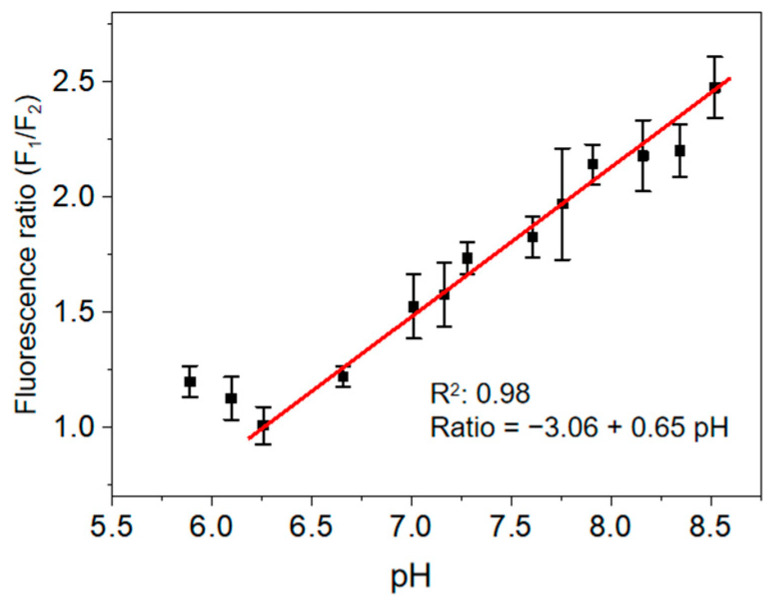
Response curve between fluorescence ratio of nano pH sensor and pH. The fluorescence signal was monitored at 513 nm (F1 was Ex 460 nm, F2 was Ex 413 nm).

Using vortex agitation, the fluorescent nanoparticles were distributed into a homogeneous and symmetrical suspension in water. The nanoparticle suspension solution was diluted a hundred times using PBS buffer and incubated at room temperature for 15 min, and afterward, the excitation and emission fluorescence spectra of the resultant solution were recorded. Pyranine is highly sensitive to pH changes due to the protonation or deprotonation of its hydroxyl group, with a pKa of approximately 7.3. This pKa, being close to physiological pH, makes pyranine particularly suitable for monitoring pH fluctuations in biological environments. The protonated and deprotonated forms of pyranine exhibit distinct absorption wavelengths, although both forms emit fluorescence at 513 nm. The sample can be excited at 460 nm (specific to the deprotonated form and pH-dependent) and at 413 nm (specific to the protonated form, serving as a pH-independent reference) [36,37]. As illustrated in Figure 3, when the pH value increased to the range of 6.25 to 8.5, an increase in fluorescence intensity (Ex 460 nm) was observed, while the pH-independent signal did not change at Ex 413 nm (Figure 3). By recording the emission intensity at 513 nm for both excitation wavelengths, the fluorescence intensity ratio (I460 (Figure 3 right)/I413) can be calculated. This dual-excitation approach facilitates ratiometric pH measurements, providing a reliable and self-calibrating method for assessing pH changes. As shown in Figure 4, the curve fitted well with a linear response in the pH 6.26–8.5 range. It is described by Equation (1) with an R^2^ of 0.98, which makes this sensor suitable for physiological applications.

Fluorescence Ratio (F_1_/F_2_) = −3.06 + 0.65pH (1)

### 3.3. Effect of Ionic Strength

**Figure 5:**
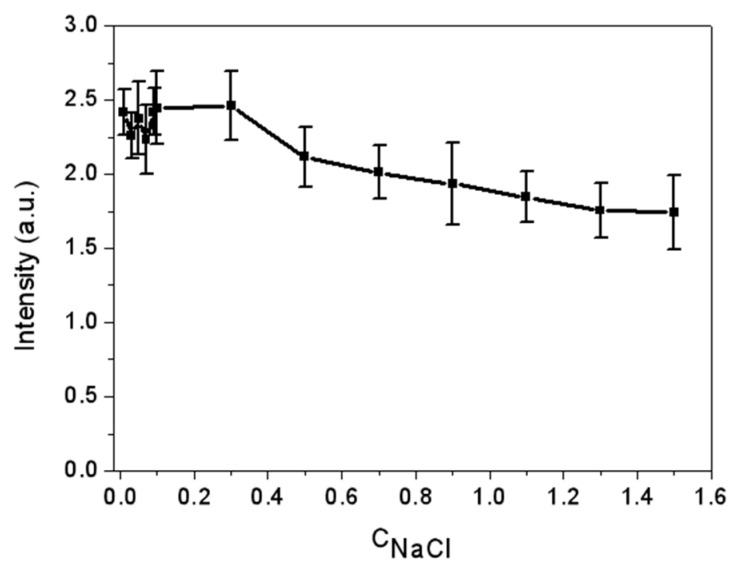
The impact of ionic strength on the fluorescence intensity of the sensor.

To examine how ionic strength affects the pH sensor, it was exposed to NaCl solutions at concentrations from 0.01 to 1.5 mol L^−1^, with pH maintained at 7.4 (Figure 5). The fluorescence intensity remained relatively stable when the ionic strength was below 0.3 mol L^−1^. Once the ionic strength reached 0.3 mol L^−1^ or higher, the fluorescence intensity of the pH sensor started to decrease as the ionic strength increased. Since the ionic strength had an effect on the pH sensor, these pH sensors could only be used at low ionic strength.

Earlier research [17] suggested that the presence of salts in the solution impacts the pH values determined by pyranine, and our findings are consistent with these results.

**Figure 6:**
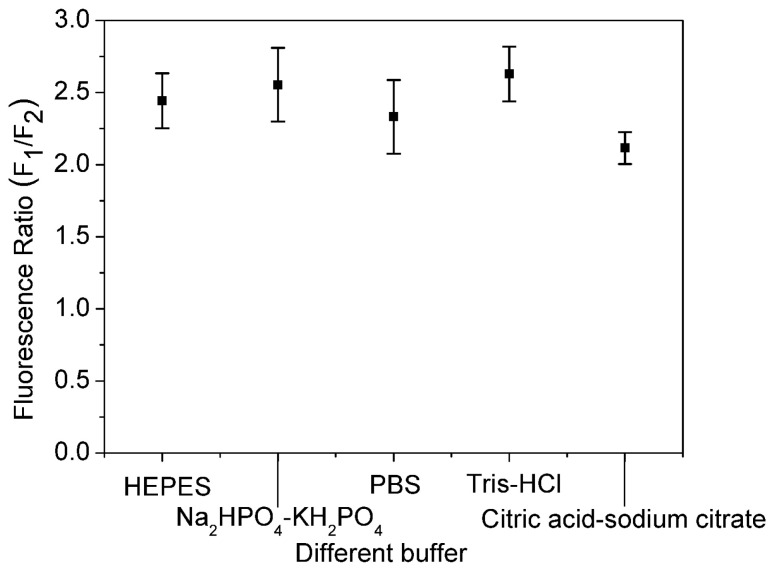
Fluorescence ratio of different pH buffers.

The pH sensor response was assessed under the influence of various buffer solutions, including 20 mM CA-Na_2_HPO_4_, PBS, Tris-HCl, Na_2_HPO_4_-KH_2_PO_4_, and HEPES. Figure 6 illustrates the variation in the pH sensor’s fluorescence intensity across different buffers. The data show that the types of buffer solution had significantly low or no influence on the pH sensor activity.

### 3.4. Stability, Reproducibility, and Reversibility of Nano pH Sensor

**Figure 7:**
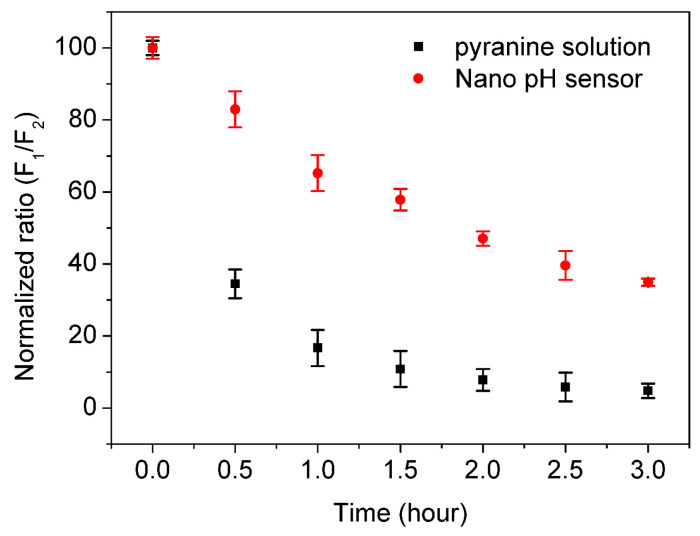
Photostability of nano pH sensor (significance test: ANOVA or *t*-test).

The photostability of fluorescent sensors is a very important characteristic with regard to their practical usage. To study the photostability of the sensor, the prepared sensor and pyranine solutions were exposed to a UV lamp (12 W) at a wavelength of 365 nm for 3 h. The decay of the fluorescent response of the sensors was insignificant, while the photostability of organic dye pyranine was quite low (Figure 7), showing that the lipid bilayer can effectively protect pyranine from photobleaching [38]. The obtained results show that the novel nano pH sensor is a highly photostable fluorescent brightener with strong potential for usage as an effective fluorescence detector.

**Figure 8:**
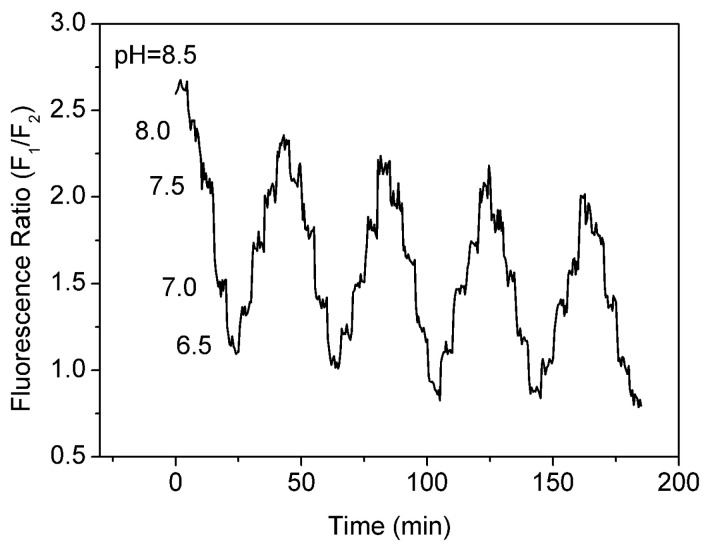
The pH-induced fluorescence ratio vs. time to evaluate the reproducibility and reversibility of the sensor.

The layer-by-layer (LbL) self-assembly technique, known for its versatility in depositing nanostructured coatings on substrates with complex geometries [39,40], has been successfully used for the deposition of many functional entities like dyes, quantum dots, and enzymes [41]. As far as we know, no reports exist on the fabrication of sensitive films using liposome-based nanoparticles. We deposited the nano pH sensor and PSS on a quartz substrate by LbL deposition and then tested the reproducibility and reversibility of the sensor. As shown in Figure 8, in each cycle, the results showed that the ratiometric signal remained consistent across repeated measurements, even as absolute intensities gradually decreased. We acknowledge that prolonged exposure to excitation light and elution by PBS with different pH values led to a gradual decrease in ratiometric fluorescence due to photobleaching and fall-off from the substrate. However, compared to commercial pyranine, our sensor design significantly improves stability by encapsulating pyranine within a cerasome hybrid structure, which enhances photostability and minimizes environmental interference, as shown in Figure 7.

### 3.5. Monitoring Intracellular pH by pH Sensors

**Figure 9:**
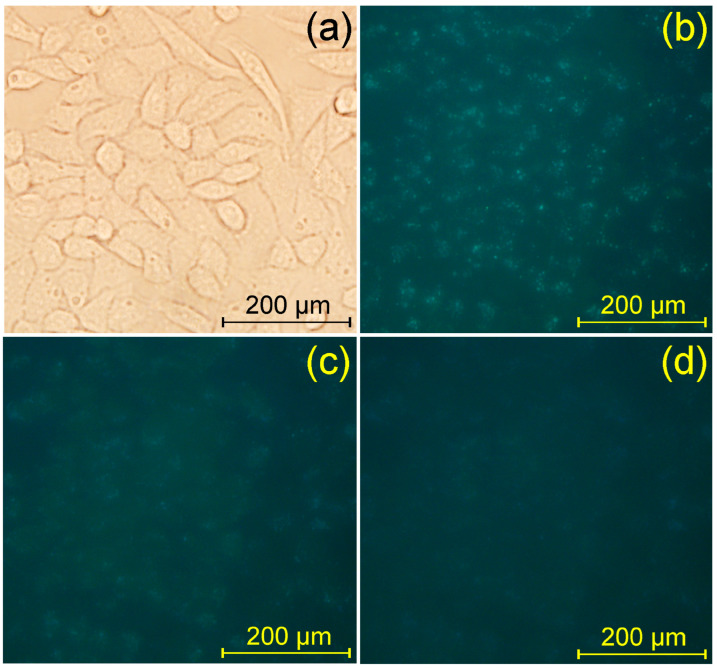
Cellular uptake of nano pH sensors by HeLa cells. (**a**) Optical image of HeLa cells without nano pH sensors. (**b**) Fluorescence image of HeLa cells after 3 h of incubation with nano pH sensors, showing the distribution of fluorescence within the cells. (**c**) Fluorescence image of the same cells after adding 10 µL of 0.1 M HCl for 10 s, illustrating the response of the sensors to a change in pH. (**d**) Fluorescence image after 30 s of HCl addition, highlighting further changes in fluorescence intensity. All fluorescence images were captured with the same exposure time..

Among intracellular species, the proton is a key target due to the central role that pH plays in various cellular processes [10,42]. To assess the suitability of the nano pH sensor for intracellular pH sensing applications, we integrated them into living cells and analyzed the effects of pH. We used HCl to change intracellular pH. The cellular uptake of the nano pH sensor is shown in Figure 9b. After adding HCl to the cell supernatant, the fluorescence intensity dropped immediately (Figure 9c,d compared to (Figure 9b)). It was very important that this lipid-based sensor could be applied for intracellular pH measurement, because highly biocompatible traditional liposomes were unstable in cells. The findings of this research suggest the potential to create a class of nanoscale, highly stable intracellular sensors that can detect not only protons (pH) but also other ionic species and physiological processes.

### 3.6. Preliminary Analytical Application

To evaluate the practicality of the proposed pH sensor, three sample types with varying pH levels (6.5, 7.5, 8.0) were tested. Samples were analyzed after incubation for 5 min. Table 1 provides a summary of the findings in comparison to those obtained with a standard glass electrode. As presented in Table 1, all results closely matched those obtained using the glass electrode method, suggesting that the sensor could enable real-time in vivo monitoring of intracellular pH.

## 4. Conclusions

We demonstrated the development and biological applications of a ratiometric fluorescence nano pH sensor for imaging intracellular H^+^ optically. The nano pH sensor, utilizing cerasome, provided the benefit of sufficient sensitivity. The proposed sensor showed remarkable stability and strong reproducibility. Moreover, the sensors exhibited significant pH dependence in buffer suspensions and in living cells. The linear response range was determined to be between pH 6.25 and 8.5. Changes in intracellular pH led to altered fluorescence intensity. Moreover, the biosensors were synthesized without difficulty, proving the fabrication method effective for practical applications. This method provided a new approach for developing a pH-sensitive fluorescent nano sensor by encapsulating indicator dye into nanoparticles. Additionally, silica nanoparticles demonstrated great biocompatibility and could be easily functionalized. Overall, these distinctive features allow this type of pH sensor to be utilized more broadly in bioanalytical applications.

## Figures and Tables

**Table 1:** Results of the evaluation of pH sensor by pH meter.

Samples	pH Meter	Proposed pH Sensor	R.S.D. (%)
1	6.50	6.85	5.38
2	7.50	7.20	4.00
3	8.00	7.79	2.63

## Data Availability

Data are contained within the article.
